# Evaluation of Differentiated Human Bronchial Epithelial Cell Culture Systems for Asthma Research

**DOI:** 10.1155/2012/943982

**Published:** 2012-01-11

**Authors:** Ceri E. Stewart, Elizabeth E. Torr, Nur H. Mohd Jamili, Cynthia Bosquillon, Ian Sayers

**Affiliations:** ^1^Division of Therapeutics and Molecular Medicine, Nottingham Respiratory Biomedical Research Unit, University Hospital of Nottingham, Nottingham NG7 2UH, UK; ^2^Division of Drug Delivery and Tissue Engineering, School of Pharmacy, University of Nottingham, Centre for Biomolecular Sciences, University Park, Nottingham NG7 2RD, UK

## Abstract

The aim of the current study was to evaluate primary (human bronchial epithelial cells, HBEC) and non-primary (Calu-3, BEAS-2B, BEAS-2B R1) bronchial epithelial cell culture systems as air-liquid interface- (ALI-) differentiated models for asthma research. Ability to differentiate into goblet (MUC5AC+) and ciliated (*β*-Tubulin IV+) cells was evaluated by confocal imaging and qPCR. Expression of tight junction/adhesion proteins (ZO-1, E-Cadherin) and development of transepithelial electrical resistance (TEER) were assessed. Primary cells showed localised MUC5AC, *β*-Tubulin IV, ZO-1, and E-Cadherin and developed TEER with, however, a large degree of inter- and intradonor variation. Calu-3 cells developed a more reproducible TEER and a phenotype similar to primary cells although with diffuse *β*-Tubulin IV staining. BEAS-2B cells did not differentiate or develop tight junctions. These data highlight the challenges in working with primary cell models and the need for careful characterisation and selection of systems to answer specific research questions.

## 1. Introduction

Asthma is a chronic respiratory condition characterised by recurrent exacerbations [1]. A feature of asthma (especially severe asthma) is airway remodelling, that is, increased smooth muscle mass, fibrosis, and excessive mucus production [2]. The epithelium plays a key role in the development of airway remodelling and inflammation as it represents the primary barrier to environmental exposures and also signals to other cell types within the context of the epithelial mesenchymal trophic unit [3, 4].

*In vitro* models using primary cells and cell lines are essential for understanding the function of the epithelium relevant to asthma. Cells are routinely cultured in submerged monolayers on a plastic substrate. In order to obtain a more physiological model, primary human bronchial epithelial cells (HBECs) may be cultured at air-liquid interface (ALI) using defined medium to drive a differentiated phenotype [5]. This model shows a pseudostratified, polarised phenotype, including ciliated and goblet cells and develops high transepithelial electrical resistance (TEER) [6, 7]. Measurement of TEER provides an indirect measure of formation of tight junctions and is often used as a marker of disruption of the epithelial layer [8].

Cultured primary HBECs from asthma and non-asthma subjects have been compared in a number of studies, to investigate intrinsic differences in the asthmatic epithelium. Epithelial cells from asthmatic patients display differential expression of genes associated with inflammation, repair, and remodelling and have been shown to differ from normal cells in culture, including increased proliferation [9] and slower repair of a mechanical wound [10, 11]. Several groups have cultured asthmatic epithelial cells at ALI, showing a less differentiated phenotype, that is, increased numbers of basal cells [12] or decreased tight junction formation [13], and differing responses to stimulation including viral infection, mechanical wounding, and cigarette smoke [12–14]. There has been some debate regarding reported differences between normal and asthmatic cells. For instance, Hackett et al. [12] report no difference in TEER between normal and asthmatic cultures, whilst Xiao and colleagues suggest that cells from asthmatic subjects show decreased TEER and disrupted tight junctions [13]. These discrepancies may reflect differences in donor profile (donors were significantly older in the Xiao study), cell source (*post mortem *donor lungs *versus* bronchial brushings), or the much greater number of subjects included in the Xiao study. Paediatric asthmatic HBECs in monolayer culture show slower repair of a mechanical wound [10, 11]. At ALI, HBECs from asthma donors show increased cytokine release in response to mechanical wounding, or viral or particulate matter exposure [12], and are more sensitive to disruption of TEER by cigarette smoke extract [13]. Another study found that whilst HBECs from normal donors showed an increased rate of wound repair in response to IL-1*β* treatment, asthmatic cells did not show this response [14]. These results may suggest that asthmatic cells at ALI have an intrinsically different phenotype and show different signalling responses to normal cells and support the utility of epithelial cell culture in asthma research.

Direct comparisons of normal and asthmatic cells allow characterisation of the asthmatic phenotype; however, they are less helpful when trying to dissect the underlying mechanisms behind epithelial changes in asthma. Normal primary bronchial epithelial cells and cell lines may be used to model various aspects of asthma. Cytokines may be added to cells in monolayer or ALI culture [15–17], whilst asthma triggers such as Derp1 or rhinovirus have been applied to the cells to mimic allergen inhalation or viral exacerbation [18, 19]. Danahay et al. treated ALI HBECs with IL-13 or IL-4, resulting in changes in permeability, suggesting that these asthma-related cytokines may contribute to a more secretory phenotype [15], whilst Wadsworth and colleagues found that addition of IL-13 and other T_H_2 cytokines led to increased MMP7 and FasL release, which may lead to epithelial damage and inflammation [16]. In another study, HBEC or BEAS-2B cells at ALI were treated with leukotriene D_4_, resulting in signalling via EGFR and release of IL-8 [17]. Overall these data demonstrate that the use of HBEC and cell line cultures can provide a unique insight into mechanisms underlying asthma and it is important to understand the strengths and weaknesses of these culture systems.

Accumulating data suggest that bronchial epithelial cells may be a viable drug target in asthma [20]. Cell culture models are used in drug development, both to assess the direct effect of potential drugs on cell function and signalling and to investigate drug uptake and metabolism [21]. Although primary cells are the gold standard, there are some disadvantages to their use including cost, limited life span, and variability between donors, passage, or experiments. Primary cells may also be more difficult to transfect or otherwise manipulate. This has led to the use of cell line systems, in both monolayer culture and at ALI. The Calu-3 cell line was established from a pleural effusion of a lung adenocarcinoma, derived from submucosal gland serous cells [22–24]. It is often used at ALI as a model system, particularly for investigations of tight junction and barrier formation [23], for instance, showing that rhinovirus infection leads to decreased TEER and increased permeability [19]. The BEAS-2B cell line, originally developed by immortalization of normal human bronchial epithelial cells using AD12-SV40 virus [25], has been less frequently used at ALI; however there is some literature using BEAS-2B in this system [17, 26]. Although these cells have been separately characterised by techniques such as immunofluorescence and TEER, no systematic comparison of primary cell and cell line culture models in this system has been reported.

The aim of the current study was to evaluate primary and non-primary bronchial epithelial cell culture systems as (ALI-) differentiated models for asthma research. We cultured primary HBECs from two donors, Calu-3, BEAS-2B, and BEAS-2B R1 (a subclone of BEAS-2B, cultured in the presence of foetal calf serum (FCS)) in their respective media at ALI. Development of TEER was measured over 28 days, RNA was collected at days 7, 14, and 21, and immunofluorescence was performed at day 28. We measured expression of a panel of differentiation (*β*-Tubulin IV, a ciliated cell marker, and MUC5AC, a goblet cell marker [27]) and tight junction/adhesion (E-Cadherin and ZO-1) markers by real-time quantitative PCR (qPCR) and confocal imaging to allow direct comparison of the phenotype of these different cell systems.

We show that although primary cells develop a differentiated phenotype, their TEER is highly variable, confirming the need to use multiple experiments and donors in primary cell systems. Calu-3 cells showed high TEER and similar expression of markers compared to primary cells, suggesting that these cells may be the most suitable model cell line for ALI experiments. Our work (1) indicates that all model systems, including primary cells, should be validated to ensure that the most suitable model is being used for a specific research question and (2) highlights the difficulties in utilising primary cells in epithelial cell research.

## 2. Materials and Methods

### 2.1. Cell Culture and ALI Differentiation

Human bronchial epithelial cells (NHBEC, Lonza, Wokingham, UK) were expanded in growth factor-supplemented medium (BEGM, Lonza) and differentiated at (ALI) at passage 3-4 in differentiation medium (BEDM) according to a previously published method [15, 28]. BEDM was composed of 50 : 50 Dulbecco's Modified Eagle's Medium (DMEM, Sigma) : BEBM (Lonza) with Lonza singlequots, excluding triiodo-L-thyronine and retinoic acid, but including GA-1000 (Gentamicin and Amphotericin-B). BEDM was supplemented with 50 nM retinoic acid at time of use. All experiments were performed using a single lot of BEBM and singlequots to avoid batch variation. Medium was used within one month of preparation, as recommended by Lonza.

Calu-3 lung adenocarcinoma cells [22] (obtained from ATCC) were cultured in Dulbecco's Modified Eagle's Medium/Nutrient Mixture F-12 Ham (DMEM/F12) (Sigma) supplemented with 10% FCS, 1% MEM non-essential amino acid solution (Sigma), and 1% Penicillin/Streptomycin (Sigma). BEAS-2B [25], a transformed bronchial epithelial cell line (gift from Dr R. Clothier, University of Nottingham), was cultured in BEGM and differentiated at ALI in BEDM. BEAS-2B R1 [29] (a subclone of BEAS-2B) (gift from Dr. R. Penn, University of Maryland, Philadelphia, PA) was cultured in DMEM supplemented with 10% FCS and 1% Penicillin/Streptomycin. All cells were cultured on 12 mm polyester Transwell inserts with a pore size of 0.4 *μ*m (Corning NY, USA). Cells were plated at 100,000 cells per insert in appropriate medium. When confluent (~3 days), cells were raised to ALI. Medium was replaced and the apical face washed with phosphate-buffered saline (PBS) every 48 hours. RNA was extracted after 7, 14, and 21 days at ALI and cells were fixed for immunostaining after 28 days at ALI.

### 2.2. Transepithelial Electrical Resistance (TEER)

The transepithelial electrical resistance (TEER) was measured in differentiating cells using an EVOM2 epithelial volt-ohm meter (World precision Instruments UK, Stevenage), over 21 to 28 days at ALI to confirm development of tight junctions. Briefly, medium was aspirated and replaced with 1 mL in the basolateral and 0.5 mL in the apical compartment. Cultures were equilibrated in the incubator for 30 minutes before measurement of TEER. Apical medium was then aspirated to restore ALI. TEER of insert and medium alone was subtracted from measured TEER and Ω·cm^2^ calculated by multiplying by the insert area.

### 2.3. Immunofluorescence of Cultured Cells

ALI cultured cells were fixed* in situ *on inserts and transferred to glass slides for visualisation. Cells were fixed using 4% formaldehyde and blocked/permeabilised with PBS, 10% goat serum, 1% BSA, and 0.15% Triton-X. Cells were incubated with appropriate primary antibodies at 4°C overnight (Table 1), and FITC or rhodamine-TRITC labelled secondary for 1 hour at room temperature before mounting in HardSet DAPI (Vector Labs). Controls were incubated with secondary antibody alone or primary isotype control antibody followed by secondary antibody. Cells were visualized using the Zeiss spinning disk confocal microscope using Volocity software (version 5.5, PerkinElmer, Cambridge, UK).

### 2.4. Quantitative PCR (qPCR)

Cultured cells were lysed and RNA was extracted using silica columns (RNeasy mini kit, Qiagen, Crawley, UK). cDNA was synthesized using Superscript II (Invitrogen, Paisley, UK) and random hexamer primers as per instructions. mRNA levels were quantified using a series of TaqMan assays (Table 2). Probes were labelled with FAM and TAMRA. qPCR was performed using TaqMan gene expression master mix (Applied Biosystems, Warrington, UK) and HPRT1 (4310890E, Applied Biosystems) endogenous control on a Stratagene MxPro3005 machine using 40 cycles of 95°C 15 sec, 60°C 60 sec. Data were normalised using the housekeeper (HPRT1) and the 2^−Δ*Ct*^ method.

## 3. Results

### 3.1. Primary Epithelial Cells and Cell Lines Develop TEER When Cultured at ALI

**Figure 1:**
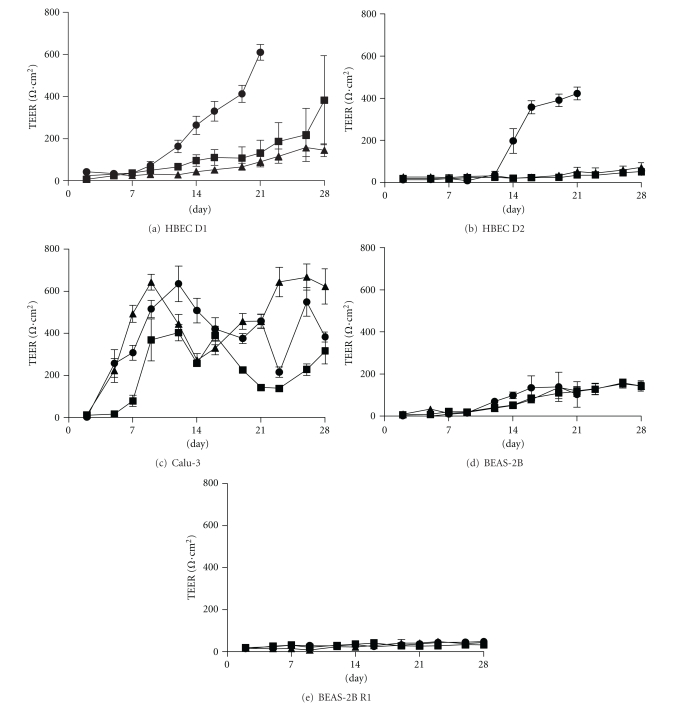
Development of transepithelial electrical resistance (TEER) in cells grown at (ALI). Different primary cells and cell lines were cultured at ALI over 21–28 days. TEER was measured every 2-3 days. Results from three separate experiments are shown for each cell line/donor, six replicates per experiment. HBEC D1 is Donor 1 and HBEC D2 is Donor 2. Error bars show standard deviation.

Primary HBECs (2 donors) and cell lines were cultured at ALI and TEER measured every 2-3 days for 21–28 days (Figure 1, Table 3). All primary cell experiments were performed at passage 3-4 from different frozen vials. Experiments performed at passage three (two in Donor 1, one in Donor 2) developed TEER >350 Ω·cm^2^, whilst experiments performed at passage four developed TEER <150 Ω·cm^2^ (Figures 1(a) and 1(b)). Calu-3 (passage 35–37) developed maximum TEER >400 Ω·cm^2^ in all experiments, reaching a peak between days 9 and 12. Thereafter, values dropped slightly before reaching a more variable plateau (Figure 1(c)). BEAS-2B reached a maximum TEER of 100–150 Ω·cm^2^ by around day 14 (Figure 1(d)), regardless of passage, whilst BEAS-2B R1 did not develop significant TEER (Figure 1(e)).

### 3.2. Primary Epithelial Cells and Cell Lines Show Morphological Differences

**Figure 2:**
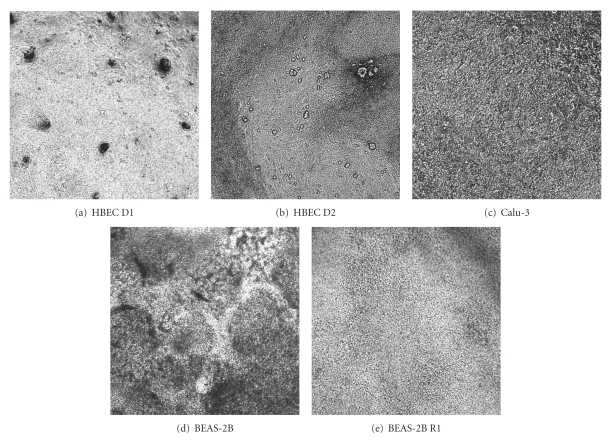
Phase contrast images of cells at ALI. Different primary cells and cell lines were cultured at ALI over 21–28 days. Phase contrast images were taken at 21 days. Representative images are from three independent experiments.

The different cells used in this study showed different phenotypes in culture. Phase contrast images give a limited indication of these differences; however gross morphological differences are present (Figure 2). Calu-3 cells took longest to become fully confluent, probably due to their tendency to form discrete colonies, unlike the other cells which form a more even monolayer. HBECs showed darker areas of denser (probably more stratified) cells and lighter, less dense areas (Figures 2(a) and 2(b)). The Calu-3 cell phenotype was more homogenous (Figure 2(c)), with increased mucus secretion apparent on washing. BEAS-2B cells consistently developed an apical layer of material which was not removed by washing (Figure 2(d)). BEAS-2B R1 cells had a very homogenous appearance, with no indication of mucus production or differentiation (Figure 2(e)).

### 3.3. Primary Epithelial Cells and Cell Lines Express Characteristic Differentiation Markers

**Figure 3:**
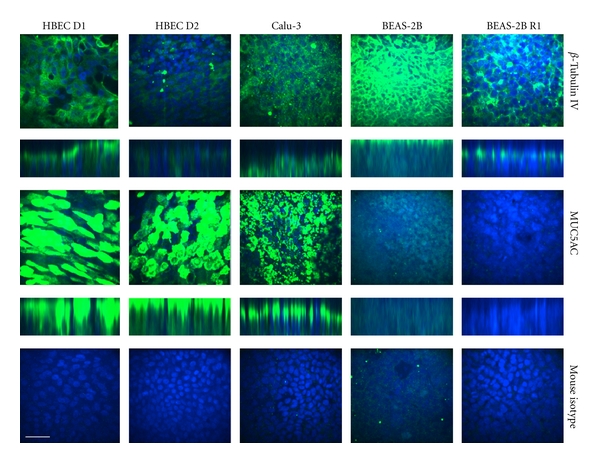
Immunofluorescent confocal imaging of differentiation markers. Localisation patterns of *β*-Tubulin IV, MUC5AC, and the Mouse IgG Isotype control at 28 days ALI were evaluated as described in the methods section. Single Z-slices are shown representing maximum intensity observed, with the corresponding Z-stack image below for *β*-Tubulin IV and MUC5AC. Scale bar represents 50 *μ*m. Representative images are from three independent experiments. HBEC images were taken from experiments with low TEER (Figure 1).

At 28 days, cells were fixed and immunostained with antibodies specific for *β*-Tubulin IV and MUC5AC (Figure 3, Table 3). Although *β*-Tubulin IV is often expressed as a cytoskeletal protein, apical expression is a commonly used marker of ciliated epithelial cells [27]. MUC5AC is expressed by goblet cells as a component of mucus. Single image slices and z-stacks are shown to give an indication of the overall level of expression and location in the cell layer (basal versus apical). As ALI culture thickness varied between cell types, the brightest image is shown in each case. These were representative of 2-3 experiments per donor or cell type. HBEC images shown for both donors are from experiments reaching low TEER; however, *β*-Tubulin IV and MUC5AC expression did not seem to reflect TEER values (data not shown). HBECs presented apical *β*-Tubulin IV expression in a subset of cells with a greater proportion of cells from Donor 1 than Donor 2 showing expression. *β*-Tubulin IV expression was observed in Calu-3 layers but staining was only apparent below the apical pole of the cells. Strong staining for *β*-Tubulin IV was obtained at the apical side of BEAS-2B cells. BEAS-2B R1 showed apical staining in a subset of cells. While both HBEC donors and Calu-3 cells was stained positive for MUC5AC expression in a subset of cells towards the apical side of the cell layer, neither BEAS-2B subtypes showed significant MUC5AC staining.

### 3.4. Primary Epithelial Cells and Cell Lines Express Tight Junction Proteins

**Figure 4:**
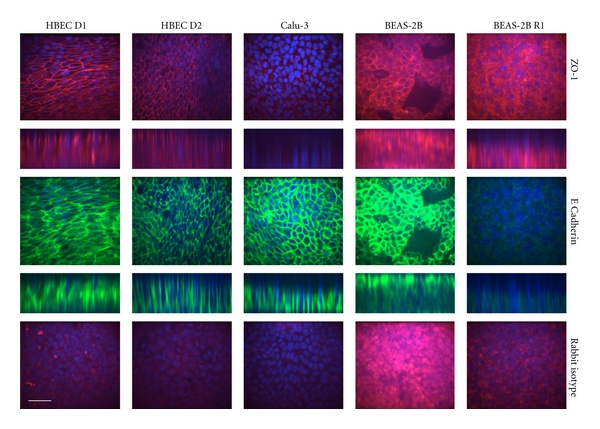
Immunofluorescent confocal imaging of tight junction proteins. Localisation patterns of ZO-1, E-Cadherin, and the Rabbit IgG Isotype control at 28 days ALI were evaluated as described in the methods section. Images shown are single Z-stack slices representing maximum intensity observed with the corresponding Z-stack image below and are of matched fields using dual staining. The corresponding Mouse Isotype control for E-Cadherin can be seen in Figure 3. Scale bar represents 50 *μ*m. Representative images are from three independent experiments. Images for HBEC Donor 1 were taken from an experiment reaching high TEER (>350 Ω·cm^2^), whilst Donor 2 images were taken from an experiment reaching low TEER (Figure 1).

Sections were costained for expression of ZO-1 (a tight junction protein) and E-Cadherin (a cell adhesion molecule and epithelial cell marker). Matched, single confocal slices and z-stacks are shown. The brightest image from each stack was chosen to allow comparison of maximum expression in each cell culture system (Figure 4, Table 3). Images are representative of 2-3 experiments per donor or cell type. Both HBEC donors showed strong staining for ZO-1 that was localised to cell membranes/cell-cell junctions. This staining may be stronger in Donor 1 (where TEER reached >350 Ω·cm^2^) than Donor 2 (where low TEER <150 Ω·cm^2^ was reached); however the difference in staining was slight, compared to the variation in TEER. Overall, ZO-1 staining was performed in five HBEC experiments and no correlation between staining and final TEER was observed (data not shown). ZO-1 expression was weaker in Calu-3 cells, despite their consistently high (>300 Ω·cm^2^) TEER, although similarly localised around cell boundaries. In both BEAS-2B subtypes, ZO-1 expression was generally diffuse; however, BEAS-2B cells showed membrane localised expression in the apical cell layer. Both BEAS-2B subtypes also showed high non-specific staining with the rabbit isotype control, suggesting that ZO-1 protein levels may be lower than they appeared. E-Cadherin expression was seen in all cells except BEAS-2B R1. In both HBEC donors and Calu-3, expression was tightly localised to the cell membrane/cell-cell junctions, whilst in BEAS-2B expression was more diffuse in the basal layer, but membrane was localised in the apical layer.

### 3.5. Expression of Differentiation and Tight Junction Markers Varies at the mRNA Level

**Figure 5:**
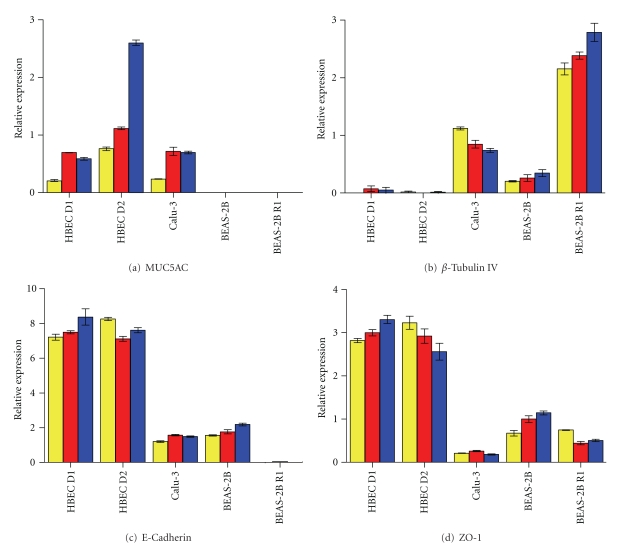
mRNA expression of differentiation and tight junction markers. Primary cells and cell lines were cultured at ALI over 21–28 days. RNA was extracted at days 7, 14, and 21 during ALI differentiation for each cell line or donor. Expression of MUC5AC (a), *β*-Tubulin IV (b), E-Cadherin (c), and ZO-1 (d) was measured. Data are normalised to the housekeeping gene HPRT1. Data are representative of two independent experiments. Error bars show standard deviation. Yellow, red, and blue bars represent expression at days 7, 14, and 21 post-ALI, respectively.

Cells were harvested for RNA at days 7, 14, and 21 during ALI differentiation and qPCR performed for MUC5AC, *β*-Tubulin IV, E-Cadherin, and ZO-1 (Figure 5, Table 3). Representative data from one of two experiments are shown. HBEC results were taken from experiments in which TEER reached >350 Ω·cm^2^ (Donor 1) and low TEER (Donor 2), whilst in replicate experiments, TEER >350 Ω·cm^2^ was reached in both donors. Expression levels of all genes were significantly different between cell types, although not between HBEC donors (*P* < 0.001 for all genes, 2-way ANOVA). These effects were conserved in a second independent experiment. Overall, no replicated trends in gene expression over time were observed.

MUC5AC mRNA was similar in HBEC and Calu-3 cells. Although expression of MUC5AC appears to increase at later time points in the experiment shown (*P* < 0.001, ANOVA), this effect was not conserved in a second independent experiment. MUC5AC mRNA was not detected in the two BEAS-2B subtypes (Figure 5(a)), consistent with immunofluorescence results. *β*-Tubulin IV expression was highest in BEAS-2B R1>Calu-3>BEAS-2B>HBEC (Figure 5(b)). This is in contrast to the immunofluorescence data where staining was lowest in BEAS-2B R1. Expression of E-Cadherin was highest in HBEC>Calu-3 and BEAS-2B>BEAS-2B R1 (not detected) (Figure 5(c)), whereas immunofluorescence was similar in HBEC and Calu-3. ZO-1 expression (Figure 5(d)) was highest in HBEC>BEAS-2B and BEAS-2B R1>Calu-3.

## 4. Discussion

We have evaluated two primary human donors of bronchial epithelial cells and Calu-3, BEAS-2B, and BEAS-2B R1 cell culture systems as ALI models of the airway epithelium for asthma research. For the first time, cell lines were directly compared to primary cells (Table 3). Using measurement of TEER [8], immunofluorescent staining, and qPCR, we have investigated formation of tight junctions (ZO-1 and E-Cadherin) as well as expression and localisation of suggested markers of ciliated (*β*-Tubulin IV) and goblet (MUC5AC) cells [27]. The main outcomes of our study are that (1) primary HBECs demonstrate a variable differentiated phenotype with the development of tight junctions and TEER showing experiment, passage, and donor variation, (2) Calu-3 cells exhibit many of the features of primary cells but have distinct differences including, for example, ZO-1 expression, and *β*-Tubulin IV localisation, although data generated were more reproducible, and (3) as anticipated, the BEAS-2B cell lines have limited differentiation capacity in ALI models. These data have implications for the use of both primary cells and cell lines for airway epithelial research in asthma.

The use of primary HBECs* in vitro* has provided insight into the potential mechanisms underlying asthma. This is exemplified by the recent findings of Xiao and colleagues [13], demonstrating that asthmatic epithelial cells at ALI show disrupted tight junctions and increased macromolecular permeability, reflecting the *ex vivo* phenotype. Another study by Hackett et al. observed an increased cytokine response to particulate matter, viral exposure, or mechanical wounding [12], demonstrating that asthmatic cells may show an aberrant inflammatory response to common environmental stimuli. Primary and cell line systems also play a role in dissecting the signalling networks involved in asthma. Normal HBECs at ALI treated with T_H_2 cytokines, for instance, show a potentially more secretory phenotype [15], whilst in HBEC or BEAS-2B cells, leukotriene D_4 _ signals via EGFR to release IL-8 [17]. It is beyond doubt that these epithelial ALI culture systems show utility in asthma research; therefore in the current study, we aimed to provide a direct comparison of a number of cell culture systems used in asthma research to help in selection of appropriate systems for specific research questions.

We measured expression of various proteins at the mRNA and protein levels as markers of differentiation. Although *β*-Tubulin IV is widely expressed in cultured cells, apical expression is often used to identify ciliated epithelial cells at ALI [27, 30], whilst MUC5AC is a mucus protein, expressed by goblet cells in the lung epithelium [31]. ZO-1 and E-Cadherin were included as markers of tight junction formation and barrier integrity. An alternative method of characterising ALI cultures is sectioning and performing histochemical analysis to confirm differentiation which gives a clearer indication of the multilayer structure (e.g. [12]). This study is limited by the use of immunofluorescence only; however we can obtain an overview of the phenotypes of different systems using this method.

In this study, we found that mRNA expression was not tightly linked to immunostaining, particularly for *β*-Tubulin IV, where HBECs showed very low mRNA levels but high protein expression. This suggests that mRNA expression may not be a good marker of functional status for these genes. Differences between mRNA and protein levels may reflect experimental or biological issues [32, 33]. In our study, samples were taken for qPCR at days 7–21 and for immunofluorescence at day 28, a limitation which may partially explain these differences. Some variation in immunofluorescence between samples may reflect different protein localisation; that is, diffuse faint staining may reflect similar amounts of protein to bright, localised staining. At the biological level, differences between mRNA and protein may reflect variation in posttranscriptional mechanisms between the cell types, such as mRNA stability or protein synthesis and turn-over.

When culturing primary cells at ALI, the choice of medium is very important, with different media delivering different degrees of stratification and cell phenotypes [5, 34]. We use “Gray's medium” (BEDM), which is reported to allow development of a pseudostratified, polarised phenotype, including ciliated and goblet cells. This was confirmed in our hands, with localised expression of E-Cadherin, MUC5AC, and *β*-Tubulin IV observed in both primary cell donors. This model is reported to develop TEER [6, 7]. We found that development of TEER was variable. TEER >350 Ω·cm^2^ was obtained in the three experiments performed at passage three, whilst TEER <150 Ω·cm^2^  was obtained at passage four, despite consistent expression of ZO-1 mRNA and protein, localised to the cell membrane/cell-cell junctions. These observations reinforce the assumption that localised ZO-1 staining is not a surrogate marker for TEER and *vice versa*, as well as the importance of passage when using primary cells. The variation seen in this study between different experiments in a single donor is indicative of the potential issues when comparing normal *versus* asthma cells. Routinely, a single experiment is performed per donor [12, 13]. It is important that these experiments are performed with cells cultured for the same period of time and in the same batch of medium to minimise experimental variation.

The Calu-3 cell-line was established from a pleural effusion of a lung adenocarcinoma, derived from submucosal gland serous cells [22–24]. Calu-3 cells are routinely cultured in FCS-supplemented media [23] and spontaneously differentiate at ALI to give significant TEER [23, 35]. These cells are reported to express ZO-1 (a tight junction protein) and E-Cadherin (an epithelial marker and cell adhesion protein). We showed development of TEER >400 Ω·cm^2^ and some expression of ZO-1 and E-Cadherin. Interestingly, although TEER was higher and more robust than in the primary HBECs, ZO-1 expression at both the mRNA and protein levels was lower, demonstrating that other tight junction proteins have a role to play in maintaining TEER. The cells expressed apical MUC5AC, as anticipated from their known secretory phenotype. However, staining for *β*-Tubulin IV expression was diffuse and not clearly located at the apical side, suggesting that villi or cilia had not formed in the Calu-3 model, in accordance with previous studies [23] that have shown that ciliated cells are sparse in the Calu-3 cell line.

The BEAS-2B cell line was originally developed by immortalization of normal human bronchial epithelial cells using AD12-SV40 virus [25]. This parental population of cells (as well as subclone S6, not used here) retains the ability to undergo squamous differentiation in response to TGF*β*1 or serum [29]. The BEAS-2B R1 line was derived from the parental population by subculture in the presence of 5% FCS. Unlike the parental cell line, these cells are induced to proliferate by serum or TGF*β*1 and have a more fusiform appearance [29]. BEAS-2B cells (S6 subclone, similar to the parental population) have previously been shown to attain TEER >100 Ω·cm^2^ at higher passage, in KGM (keratinocyte growth medium, Clonetics) when supplemented with calcium [26], or when grown in BEGM [36] or Laboratory of Human Carcinogenesis (LHC) serum-free medium [37]. We used BEDM to drive BEAS-2B towards a differentiated phenotype. These cells attained the reported TEER of >100 Ω·cm^2^. At ALI, these cells developed an apical layer which stained strongly for *β*-Tubulin IV and showed localised ZO-1 and E-Cadherin staining. However, staining was fainter and more diffuse in the basal layer. It may be the presence of this apical layer that increases the TEER, rather than tight junction formation throughout the culture model. These cells did not express MUC5AC.

There is no literature regarding the use of the BEAS-2B R1 cell line at ALI; therefore these cells were included essentially as a negative control, cultured in DMEM with 10% FCS. As anticipated, these cells did not develop TEER and expressed minimal levels of E-Cadherin at the RNA and protein level. The cells show apical *β*-Tubulin IV expression, but no MUC5AC or localised ZO-1 expression. The reduced E-Cadherin expression of this cell line suggests that they may have developed a more mesenchymal phenotype by culturing in the presence of FCS. 

## 5. Conclusions

Normal and asthmatic primary bronchial epithelial cells and cell lines are widely used in asthma research. ALI models are used to attempt to more closely replicate the *in vivo* situation. We have evaluated primary bronchial epithelial cells from two donors and three cell lines in an ALI model with respect to various markers of differentiation. Although primary cells are regarded as the most physiologically relevant, they exhibit a high degree of variability between donors, experiments, and passage, particularly with respect to development of TEER. Primary cells are costly and therefore unsuitable for large scale experiments such as drug screening; they also have a finite lifespan and may be difficult to manipulate. Cell lines may, therefore, present an attractive alternative model. We found that Calu-3 cells develop a high TEER and have a pattern of expression of epithelial markers similar to primary cells. Although frequently used in monolayer culture, the two BEAS-2B cell lines did not perform well in the ALI model, showing poor TEER and lacking expression of epithelial differentiation markers.

This work underlines the importance of using a well-characterised model, suitably validated for the outcomes of interest in any experiment. Importantly, this study highlights some of the challenges ahead characterising primary human airway epithelial cells from asthma and control donors accounting for the inter- and intradonor variability identified in the current study.

## Figures and Tables

**Table 1:** Antibodies used for immunofluorescent staining of cultured cells.

Target	Antibody	Dilution	Secondary
*β*-Tubulin IV	Sigma T7941	1 : 500	Goat anti-Mouse FITC (Sigma F0257) 1 : 100
MUC5AC	Abcam AB3649	1 : 250	
E-Cadherin	Millipore MAB3199Z	1 : 500	
Mouse IgG	Isotype Sigma M7894	1 : 463	
ZO-1	Invitrogen 40-2200	1 : 125	Goat anti-Rabbit Rhodamine TRITC (Stratech 111-025-003) 1 : 100
Rabbit IgG	Isotype Abcam AB27472	1 : 1	

**Table 2:** Primers and TaqMan probes used for qPCR assays.

Target	Primers	Probe
*β*-Tubulin IV	AGATCGTGCACCTGCAGG	CCAGTGCGGCAACCAGATCGG
	CATGGTATGTGCCTGTGG	
MUC5AC	TACTCCACAGACTGCACCAACTG	TGTGCTTGGAGGTGCCCACTTCTCAA
	CGTGTATTGCTTCCCGTCAA	
E-Cadherin	CCCACCACGTACAAGGGTC	CGAGGCTAACGTCGTAATCACCACACTGA
	CTGGGGTATTGGGGGCATC	
ZO-1	GCGGTCAGAGCCTTCTGATC	ACTCGCCGCAGCAGCCAAGCAAT
	CATGCTTTACAGGAGTTGAGACAG	

**Table 3:** A summary of outcomes for different cell types. For *β*-Tubulin IV and MUC5AC, “localised” expression refers to expression in a subset of cells. For E-Cadherin and ZO-1, expression was “localised” to the cell boundaries. *staining was similar to isotype control. HBEC D1 is Donor 1 and HBEC D2 is Donor 2.

		HBEC D1	HBEC D2	Calu-3	BEAS-2B	BEAS-2B R1
TEER		variable	variable	high	low	none

*β*-Tubulin IV	mRNA	low	low	high	mid	high
	protein	mid	mid	mid	high	mid
		localised	localised	diffuse	localised	localised

MUC5AC	mRNA	high	high	high	none	none
	protein	high	high	high	low	none
		localised	localised	localised	diffuse	

E-Cadherin	mRNA	high	high	mid	mid	none
	protein	high	high	high	mid	low
		localised	localised	localised	part localised	diffuse

ZO-1	mRNA	high	high	low	mid	mid
	protein	high	high	low	mid*	low*
		localised	localised	localised	part localised	diffuse
